# An Atypical Case of *Bartonella henselae* Osteomyelitis and Hepatic Disease

**DOI:** 10.1155/2018/2750275

**Published:** 2018-04-05

**Authors:** Dionna M. Mathews, Katie M. Vance, Pamela M. McMahon, Catherine Boston, Michael T. Bolton

**Affiliations:** ^1^Our Lady of the Lake Children's Hospital, Baton Rouge, LA, USA; ^2^Division of Academic Affairs, Our Lady of the Lake Regional Medical Center, Baton Rouge, LA, USA; ^3^Pediatric Hematology/Oncology, Our Lady of the Lake Children's Hospital/St. Jude Affiliate Baton Rouge, Baton Rouge, LA, USA; ^4^Pediatric Infectious Diseases, Our Lady of the Lake Children's Hospital, Baton Rouge, LA, USA

## Abstract

*Bartonella henselae* is a Gram-negative bacterium and the causative agent of cat scratch disease (CSD). Atypical presentations of *B. henselae* that involve the musculoskeletal, hepatosplenic, cardiac, or neurologic systems are rare. In this case report, we describe a case of *B. henselae* osteomyelitis involving bilateral iliac bones complicated by hepatic lesions in a 12-year-old immunocompetent female patient. Although *B. henselae* is a rare cause of osteomyelitis, it should be considered when patients who present with fever, pain, and lymphadenopathy do not respond to routine osteomyelitis therapy.

## 1. Introduction

*Bartonella henselae*, a Gram-negative bacterium, is the causative agent of cat scratch disease (CSD) that typically involves the mononuclear phagocyte system and presents as local lymphadenopathy, often accompanied by fever [1]. *B. henselae* infections are thought to occur when a human is bitten or scratched by an infected cat [2–4] and may be transmitted by cat fleas or by an infected cat licking the nonintact skin of a human [5–7]. IgM is often elevated only briefly and is commonly normal during the course of the disease [8]. IgG titers greater than 1 : 256 are typically indicative of previous or active disease [6]. Children and teenagers make up approximately 80% of patients diagnosed with CSD [2]. Although infrequent, *B. henselae* can affect almost every organ system after hematogenous, lymphatic, or contiguous spread.

*Bartonella* osteomyelitis most frequently occurs in the spine. The pelvic girdle is the most common site of *Bartonella* osteomyelitis outside of the spine and occurs in 42% of all nonspinal cases [3]. *Bartonella* osteomyelitis typically presents as tenderness or pain in the affected area [4]. Magnetic resonance imaging (MRI) or radionuclide bone scanning is often used to diagnose osteomyelitis. Clinicians must rely on serologic testing, polymerase chain reaction testing (PCR), or Warthin–Starry silver staining to identify *B. henselae* as the causative organism because it does not grow in culture [3, 4]. We present a case of *B. henselae* osteomyelitis involving bilateral iliac bones complicated by hepatic lesions in an immunocompetent patient.

## 2. Case Presentation

A previously healthy 12-year-old female presented to the emergency department 4 days after completing a 3-day course of trimethoprim/sulfamethoxazole prescribed for a urinary tract infection. She complained of a 9-day history of fever and 3-day history of left hip pain associated with joint movement. The patient denied trauma, erythema, or swelling in the area of pain, but exposure history uncovered prolonged cat contact. Her initial exam revealed full range of motion of all extremities though she exhibited tenderness to palpation of her left hip. The remainder of the exam was unremarkable, including absence of any lymphadenopathy, bruising, erythema, or edema of the affected joint.

The patient's initial workup was significant for normocytic anemia (hemoglobin of 11.9 g/dL and hematocrit of 34.5%) and normal liver function tests. She was noted to have elevated inflammatory markers—an erythrocyte sedimentation rate greater than 120 mm/h and C-reactive protein of 68.6 mg/L. Urinalysis showed small amounts of leukocyte esterase and bacteria but only 0–5 white blood cells (WBCs). Urine and blood cultures were obtained and showed no bacterial growth.

**Figure 1:**
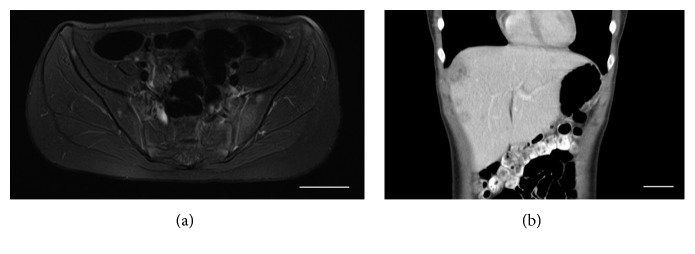
MRI and CT of patient's *Bartonella* bone and hepatic lesions. (a) An MRI obtained of the patient's pelvis shows small, scattered lesions throughout the iliac architecture. (b) A CT of the patient's abdomen shows hepatic lesions indicative of *B. henselae*. Bar, 5 cm.

An X-ray of the hip showed no acute abnormalities; however, a subsequent MRI showed a small left sacroiliac joint effusion with mild marrow edema that was concerning for infectious or inflammatory sacroiliitis (Figure 1(a)). Scattered small round lesions replaced bone marrow throughout the iliac architecture.

The patient was admitted with a working diagnosis of septic arthritis and received clindamycin intravenously. Joint fluid obtained via CT-guided aspiration revealed clear fluid with 15 WBCs, 200 red blood cells, and sterile cultures. Antibiotics were discontinued given that these findings were inconsistent with pyogenic arthritis, and she was improving clinically. Concern for possible leukemia based on the abnormal bony lesions led to an oncologic evaluation, including a chest X-ray, peripheral blood smear, and measurement of lactate dehydrogenase and uric acid, all of which were within normal limits.

Due to recrudesce of fever following the discontinuation of antibiotics, Epstein–Barr virus, cytomegalovirus, and *B. henselae* antibodies were measured. The patient's fever again declined without directed therapy, and she was discharged home with a diagnosis of transient synovitis.

The patient had intermittent low-grade fevers the week following her discharge. Her *B. henselae* titers were suggestive of recent infection (IgG > 1 : 1024, IgM negative). Due to known hepatic involvement of *B. henselae* in association with disseminated *Bartonella* disease, imaging (abdominal ultrasound followed by CT scan; Figure 1(b)) was performed and showed multiple liver lesions thought to be consistent with disseminated *B. henselae* infection. Cytopathology of the biopsied sample taken from the hepatic lesions revealed scars consistent with resolving *Bartonella* liver lesions. While we observed inflammation, we did not observe the granulomatous inflammation that is a more classic sign of CSD. PCR of the lesions was negative for *B. henselae*. Further oncologic evaluations of the hepatic lesions were negative, as was a QuantiFERON–Gold test for tuberculosis.

The patient had clinical resolution after a 6-week azithromycin regimen. An MRI of the patient's pelvis at the end of therapy showed near complete resolution of the abnormalities in the iliac bones, which were fully resolved in an MRI obtained one year later. Furthermore, a CT of the patient's abdomen obtained at the end of therapy revealed that the liver lesions were decreasing in size, consistent with resolving infection. The patient's inflammatory markers also were normal.

## 3. Discussion

CSD often goes unrecognized due to nonspecific signs and symptoms and the disease's usual self-limiting natural course. Osteomyelitis caused by *B. henselae* is rare (observed in 0.1%–0.3% of CSD patients) but has long been recognized to occur in both immune compromised and immune competent patients [9, 10]. Although PCR is generally considered more sensitive for the diagnosis of *B. henselae* infection than is serology, its sensitivity is still not very good, with a reported range from 43% to 76% [4]. Indeed, we were unable to detect *B. henselae* from hepatic sampling via PCR. Insufficient fluid volume precluded testing on the joint sample, and blood was not sampled for PCR detection. The diagnosis of disseminated *B. henselae* was based on significantly elevated titers, radiographic findings, and epidemiologic history.

An oncologic process often is included in the differential diagnoses of patients who present with CSD, as *B. henselae* can produce osteolytic bone lesions that may be misdiagnosed as tumors [3, 4]. While an oncologic process was considered in the differential diagnosis of our patient's bony lesions, our patient's symptoms, combined with an unremarkable corroborative diagnostic evaluation and resolution, excluded a malignancy.

*Bartonella* osteomyelitis most commonly involves the axial skeleton, with several cases reported in the vertebrae, skull, sternum, ribs, and pelvis [1, 11, 12]. While Rozmanic et al. documented vertebral and unilateral ilial involvement, our case involves bilateral iliac infection with hepatic lesions and concurrent lack of transaminitis of an immunocompetent child [13]. The pathogenesis of *Bartonella* osteomyelitis is not well understood. In most cases of *Bartonella* osteomyelitis, the bone lesion occurs at a distance from the involved lymph nodes, suggesting hematogenous or lymphatic spread of the infection [4, 10, 14]. *Bartonella* osteomyelitis usually presents subacutely in the axial skeleton, as we observed in our patient, unlike hematogenous osteomyelitis, which presents acutely and typically involves the appendicular skeleton [9].

CSD typically resolves within weeks to months regardless of antimicrobial therapy due to its self-limiting nature. However, studies show that various antibiotics have been used to treat the multitude of clinical manifestations produced by *B. henselae* [1]. A single clinical trial that showed mild-to-moderate hastening of initial recovery with azithromycin therapy supports its use as a first-line agent for lymphadenopathy [15]. However, azithromycin's efficacy in treating patients with atypical CSD, including patients with osteomyelitis and hepatosplenic involvement, has yet to be evaluated [4, 15–17]. Our patient's history of significant cat exposure, elevated anti-*B. henselae* IgG titer, and radiographic findings in bone and liver supported the diagnosis of CSD. We treated our patient with azithromycin given the severity of her symptoms and the evidence of multisystem involvement. After a prolonged course of therapy, she appears to have made a full recovery.
